# The Role of Knowledge on Nursing Students’ Attitudes toward Organ Donation: A Cross-Sectional Study

**DOI:** 10.3390/healthcare11243134

**Published:** 2023-12-09

**Authors:** Mai B. Alwesmi, Amirah Ibrahim Alharbi, Amjad Abdullah Alsaiari, Asalah Emad Abu Alreesh, Bashair Abdullah Alasmari, May Awad Alanazi, May Khalil Alanizi, Nouf Mohammed Alsaif, Razan Mohammed Alanazi, Sheikhah Abdullah Alshdayed, Yasmine Alabbasi

**Affiliations:** 1 Department of Medical-Surgical Nursing, College of Nursing, Princess Nourah bint Abdulrahman University, P.O. Box 84428, Riyadh 11671, Saudi Arabia; mbalwesmi@pnu.edu.sa; 2 College of Nursing, Princess Nourah bint Abdulrahman University, P.O. Box 84428, Riyadh 11671, Saudi Arabia; 439000815@pnu.edu.sa (A.I.A.); 439000334@pnu.edu.sa (A.A.A.); 439005185@pnu.edu.sa (B.A.A.); 439003494@pnu.edu.sa (M.A.A.); 439005548@pnu.edu.sa (M.K.A.); 439003449@pnu.edu.sa (N.M.A.); 439001737@pnu.edu.sa (R.M.A.); saalshdayed@pnu.edu.sa (S.A.A.); 3 Department of Maternity and Child Health Nursing, College of Nursing, Princess Nourah bint Abdulrahman University, P.O. Box 84428, Riyadh 11671, Saudi Arabia

**Keywords:** nursing students, knowledge, attitude, organ donation, organ transplant, nursing education

## Abstract

Background: Nursing students constitute the future nursing workforce; their knowledge can readily influence potential donors’ decisions on organ donation. This study aimed to assess nursing students’ knowledge of organ donation, determine its impact on their attitude, and identify relevant factors. Methods: A cross-sectional comparative study assessed the level of knowledge and attitude toward organ donation among nursing students using the following two questionnaires: (1) knowledge about organ donation and (2) attitudes toward posthumous organ donation. Non-probability convenience sampling was employed to collect data. Results: A total of 278 nursing students demonstrated a low level of knowledge on organ donation, with a score of 6.43 out of 15. This influenced their attitude toward organ donation (χ^2^ (3) = 33.91, *p* < 0.001). Nursing students who were willing to donate their organs showed higher knowledge (7.33 ± 3.23) compared to those who were not willing to donate their organs (5.21 ± 3.09), *p* < 0.001. Registered donors had higher knowledge (8.52 ± 2.99) than non-donors (5.80 ± 3.17), *p* < 0.001. Conclusions: Even though nursing students typically favor organ donation, findings have revealed a low knowledge score. Therefore, it is necessary to improve knowledge of organ donation through nursing curricula and research, which could potentially increase the number of donors among future nursing students and, by extension, the broader population.

## 1. Introduction

The demand for organ transplantation has steadily increased over the years, whereas the supply of organs has plateaued, leading to severe organ scarcity and longer waiting periods for critically ill patients [1]. Although the lack of transplantable organs is a global issue, it is particularly evident in countries such as the Kingdom of Saudi Arabia (KSA), where more than 17,000 individuals are currently on the organ transplant waiting list [2].

The majority of transplanted organs originate from cadavers. However, as the need for organs continues to increase, it is crucial to explore alternative methods to bridge the gap between the increasing number of patients waiting for organ transplants and the limited availability of cadaveric organs, as well as the rising number of people passing away while on waiting lists. For instance, for many individuals with end-stage renal disease, kidney transplantation, particularly live-donor kidney transplantation, is the best treatment option [3]. Donations by living people include a direct donation to a loved one or friend, a non-direct donation to the general pool to be transplanted into the person at the top of the waiting list, and a direct donation to a stranger with no emotional connection. Nevertheless, living organ donation raises an ethical conundrum because it involves endangering a healthy person to prolong or improve the patient’s life [4].

Donations after brain death remain the primary source of organ transplantation. Brain death occurs when all brain processes cease, resulting in irreversible brain damage. After brain death, family members may make altruistic decisions to donate the organs of the patient [5]. Most organs transplanted in the United States are procured after the determination of death based on the neurological criteria of brain-dead donors [6]. Organ donation can also occur after circulatory death, which is characterized by the permanent loss of consciousness and brainstem function. However, organs from circulatory-dead donors, especially in liver transplantation, perform worse than those from brain-dead donors [7].

Many factors influence people’s decisions to donate organs. Although religious objections have historically been the most prevalent cause of refusal to donate [1], recent data acquired through questionnaires revealed that knowledge is a main causal factor [3,4]. It significantly affects a person’s decision to donate their organs after death [8]. Younger people with higher educational backgrounds are frequently more charitable than older and less educated people [9]. Therefore, several initiatives have been launched to increase knowledge on organ donation, such as public awareness campaigns establishing national-level organ donor registries and transplantation coordinators [10]. In KSA, the “Tawakkalna” application, in cooperation with the Saudi Center for Organ Transplantation, awards medals as moral support and social recognition to organ donors, with gold medals awarded to donors of all organs, silver medals to donors of two or more organs, and bronze medals to donors of one organ [11]. However, the impact of this initiative on the public awareness of donations has not been sufficiently investigated.

Healthcare professionals, particularly nurses, may significantly impact potential donors’ decisions as they are often the first point of contact with the patients and their families [12,13]. Nurses remain with patients until their moment of death; hence, their role is crucial when talking to the family about organ donation when the patient qualifies as a donor [14]. While approximately 90% of people support organ donation, less than 55% of potential donors can provide their consent because nurses have noted the difficulty of initiating the subject of organ donation [15]. Moreover, when nurses recognize the importance of brain-dead organ donation and have favorable attitudes toward the donation process and its role, they can facilitate it [16]. Therefore, considering the knowledge and methods used by medical personnel in obtaining organs for transplantation is extremely crucial [17].

Despite the increasing emphasis on the importance of nurses’ roles in promoting organ donation, nursing curricula still lack specific content on knowledge and attitudes about organ transplantation and donation, as well as nurses’ roles in these processes [13,14]. Undergraduate nursing students demonstrated a lack of understanding of diagnostic tests and comorbid variables associated with brain death, and their attitudes toward organ donation were diverse and ambiguous [18]. It remains unclear whether their knowledge has an impact on their attitude toward organ donation.

The aims of the present study were threefold. Firstly, we aimed to assess the knowledge of nursing students on organ donation. This included identifying potential donors, transplantable organs, and appropriate criteria for organ donation and transplantation. Secondly, we investigated the role of knowledge on nursing students’ attitudes toward organ donation. Thirdly, we explored the factors affecting nursing students’ knowledge and attitudes toward organ donation, such as academic performance and the concept of a donation medal.

## 2. Materials and Methods

### 2.1. Design

A cross-sectional comparative study design was utilized, which could yield valuable insights into the population characteristics of interest and allow comparisons to identify the role of relevant factors. The knowledge and attitudes of university nursing students toward organ donation were measured using two self-administered questionnaires. Demographic comparisons were then conducted to explore the factors affecting their knowledge and attitudes toward organ donation.

### 2.2. Research Tools

The following two questionnaires were adopted to conduct the survey: (1) knowledge about organ donation [19] and (2) attitudes toward posthumous organ donation [20]. These questionnaires were specifically developed to address this topic among nursing and medical students [19,20], which made them suitable to achieve the current study aims. Necessary permissions were obtained from the original authors [19,20].

Marván et al. [19] developed the knowledge of the organ donation questionnaire, comprising a set of 12 questions, including topics such as identifying the types of donors (living, brain-dead, or cardiopulmonary arrest donors), the most suitable donor type for organ extraction, and a list of eight organs or tissues that can be donated. In addition, the questionnaire included nine true, nine false, and “I do not know” statements. A global knowledge score of 15 was assigned based on correct answers [19].

Jasso K. et al. [20] described the attitudes toward the posthumous organ donation questionnaire, using a five-point Likert scale consisting of responses ranging from (1) strongly disagree, (2) disagree, (3) neutral, (4) agree, and (5) strongly agree. It comprised 21 items categorized into the following three subscales: one positive subscale, indicating a favorable attitude, and two negative subscales, indicating an unfavorable attitude and distrust. The favorable attitude scale includes seven items, these are: item 1: “It is necessary to promote the culture of organ donation when dying.”, item 4: “Donating organs helps improve the quality of life of others”, item 8: “It is satisfying that the organs can help others, even if they’re strangers.”, item 11: If a member of my family decides to donate his/her organs before he/she dies, I would do whatever was necessary to make the donation happen.”, item 15: “Organ donation gives hope to other people.”, item 18: “ When we die, our organs can be used to make other people recover their health.” and item 21: “Organ donation is an act of love towrd others”. The unfavorable attitudes scale includes nine items, these are: item 2: “Organ donation is against my religion”, item 5: “Prolonging life through organ donation is artificial.”, item 6: “I am afraid that after I die, my organs will be donated.”, item 9: “It is unpleasant to think that by donating organs the body is incomplete”, item 12: “When someone is grieving about a family member’s death, it’s disrespectful to ask that his/her organs be donated.”, item 13: “ Organ donation interrupts the natural process of dying.”, item 16: “I’m opposed to donating my organs because they belong to one person only.”, item 19: “Organ donation shows a lack of respect for the donor’s body.”, item 20: “I feel that if my organs are donated when I die, I would not rest in peace”. The distrust attitude scale consisting of five items that reflect opponents of organ donation and lack of faith in organ donation and transplantation ethics, these are: item 3: “I feel distressed thinking that if I have an accident and I am a donor, I will be poorly treated.”, item 7: “I distrust the institutions where organ transplants are performed”, item 10: “People make a business of organ donation.”, item 14: “I am worried about not really being dead when surgery is performed for organ donation.” and item 17: “I refuse to donate my organs when I die because there is a lot of corruption in the process.” [20]. The questionnaire’s authors reported the high reliability of each subscale at 0.85, 0.84, and 0.69, respectively [20].

In addition, the survey included questions regarding the willingness to donate, donation registration status, and the type of registered donors according to the Saudi organ donation system.

### 2.3. Study Population

This study focused on undergraduate nursing students from their first year to their internship year who were enrolled at Princess Nourah bint Abdulrahman University in Riyadh, Saudi Arabia, between 12 March 2022 and 30 May 2022.

### 2.4. Sample Size

The sampling method used was non-probability convenience sampling. Considering the small study population, which consisted of 948 nursing students, a 90% confidence level with a 5% margin of error was statistically acceptable. Therefore, the calculated sample size was 212 nursing students.

### 2.5. Data Collection

Once ethical approval was obtained, the survey was administered to five randomly selected nursing students to evaluate its clarity, applicability, and feasibility. The students confirmed the clarity of the survey; therefore, no modifications were required. The survey was then uploaded to Google Docs and distributed via email to nursing students on 12 March 2022. This was followed by two reminders until the sample size was achieved on 30 May 2022.

### 2.6. Inclusion and Exclusion Criteria

This study encompassed Saudi full-time undergraduate nursing students from the first to fifth (internship) levels at Princess Nourah bint Abdulrahman University. Students who did not meet the inclusion criteria were excluded.

### 2.7. Variables to Study

The variables were determined to achieve the aims of the present study, which were mainly to assess the knowledge and attitudes of nursing students toward organ donation and to explore relevant factors. The variables were as follows: knowledge and attitudes toward organ donation, a willingness to donate organs, donation registration status, donation medals, academic level, academic performance, and knowledge level.

### 2.8. Data Analysis

Data analyses were conducted using IBM SPSS Statistics software, version 28.0. Cronbach’s alpha test was utilized to assess the internal consistency of the questionnaires’ items, denoting the degree to which they functioned as a cohesive unit. The reliability of the knowledge questionnaire was estimated to be 0.76, while the attitude questionnaire had a reliability estimate of 0.70. The normality of the dataset was evaluated using the Shapiro–Wilk test and the equality of variance between the groups was assessed using Levene’s test. Demographic comparisons involving variables that followed a normal distribution were conducted using the independent sample *t*-test for two-group comparisons or one-way analysis of variance (ANOVA) for more than two groups, followed by Tukey’s post hoc test. Comparisons involving variables that did not follow a normal distribution were performed using the Mann–Whitney U test for two-group comparisons or the Kruskal–Wallis H test for more than two groups, followed by the Dunn–Bonferroni post hoc test. A *p*-value ≤ 0.05 was considered statistically significant, a *p*-value ≤ 0.005 was considered highly significant, and a *p*-value ≤ 0.001 was considered very highly significant for the results.

### 2.9. Ethical Consideration

This study was approved by the Institutional Review Board of Princess Nourah Bint Abdulrahman University (PNU) (IRB Log Number: 22-0145). A convenient sample of eligible participants was voluntarily invited via email announcements from the Deanship of Scientific Research of PNU. Their consent was obtained at the beginning of the online survey with an emphasis on their right to leave the survey at any time. The data were anonymized and encrypted using the computer of the first author.

## 3. Results

### 3.1. Demographic Characteristics

A total of 278 nursing students from Princess Nourah bint Abdulrahman University participated in this study. Most students (86%) fell within the age group of 18 to 22 years old, with only 1.5% aged above 28 years. There were roughly even numbers of students in each academic level as follows: first year (18.7%), second year (18%), third year (21.2%), fourth year (22.3%), and fifth year (internship) (19.8%). Regarding academic performance, more than half of the students (53.2%) had very good academic performance, 29.1% had excellent academic performance, 12.9% had good academic performance, and 4.7% had acceptable academic performance. Most students had never received training in organ donation (98.6%). Although 57.6% expressed a willingness to donate their organs, only 23% were registered as donors. The “Tawakkalna” application, in cooperation with the Saudi Center for Organ Transplantation, awards medals as moral support and social recognition to organ donors, with gold medals awarded to donors of all organs, silver medals to donors of two or more organs, and bronze medals to donors of one organ [11]. Of the registered donors, 14.4% received gold medals, 4.7% received silver medals, and 4% received bronze medals (Table 1).

### 3.2. Knowledge about Organ Donation

The nursing students’ overall knowledge score regarding organ donation was below average, with a mean score of 6.43 out of 15 (Table 2). The frequencies of correct and incorrect answers are summarized in Table 3. While most nursing students were able to identify the different types of donors (Item 1), more than half were unaware of the type of donor from which most organs are extracted (Item 2). Regarding organ transplantability, the majority of nursing students were aware of the skin, liver, pancreas, and brain but were unaware of the bone, chondrocytes, blood vessels, and heart (Item 3). Approximately 90% of nursing students acknowledged that transplant candidates died due to organ shortages (Item 4). However, only half believed that it was possible to have an open-casket funeral after postmortem organ donation (Item 5). More than two-thirds of nursing students were aware that brain death is the irreversible cessation of all brain functions (Item 7), and altruistic donation involves a non-family member as the recipient (Item 10). Conversely, most nursing students were unaware that organ donation is considered a blessed act in Islam (Item 6), brain death is a legal death criterion (Item 8), there is no chance of recovery (Item 9), there is no age limit for organ donation (Item 11), and cardiovascular disease does not prevent organ donation (Item 12).

To investigate the relationship between knowledge and attitudes toward organ donation, participants were categorized according to their level of knowledge based on the following quartiles: high (>9) (29.9%), moderate (6–8) (33.1%), low (4–5) (17.6%), and poor (<4) (19.4%) (Table 2).

### 3.3. Attitude toward Organ Donation

The attitude scale toward organ donation included one positive subscale, indicating a favorable attitude, and two negative subscales, indicating unfavorable attitudes and distrust (Table 3). The mean overall favorable attitude score for nursing students was 4.25 (SD = 0.78), indicating a generally favorable attitude toward organ donation. However, they demonstrated neutral unfavorable attitudes (mean = 2.40, SD = 0.88) and distrust (mean = 2.60, SD = 0.92).

### 3.4. Demographic Comparisons

#### 3.4.1. Willingness to Donate

**Figure 1:**
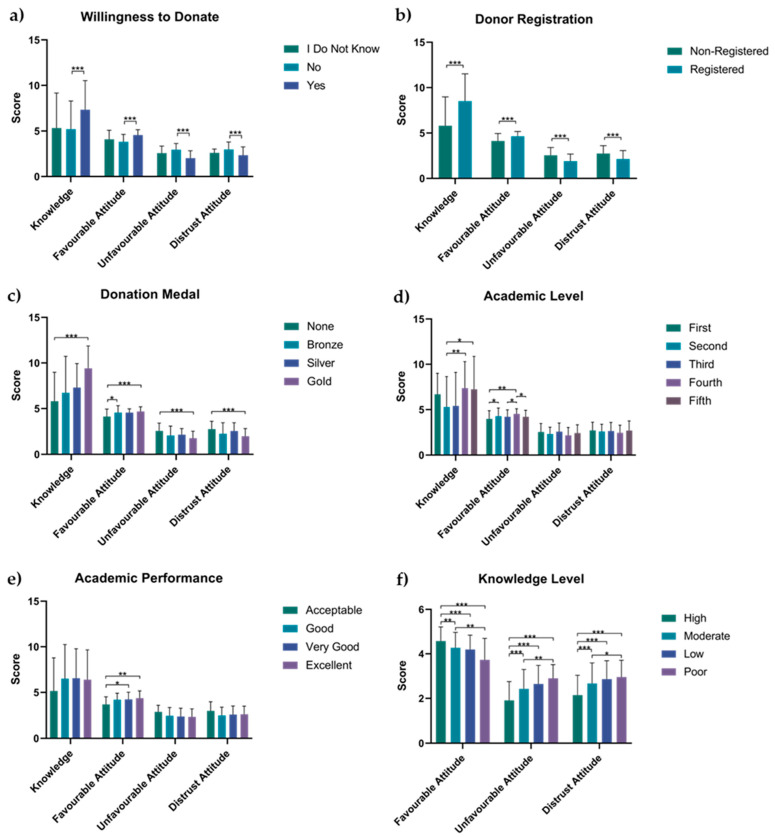
Group comparisons on overall knowledge, favorable attitude, unfavorable attitude, and distrust scores among nursing students who were willing to donate their organs and those who were not (**a**); nursing students who registered to donate their organs and those who did not (**b**); nursing students who received gold (agreed to donate all organs), silver (indicate accepting to donate two or more organs), bronze (awarded to donors of one organ), or no donation medal (**c**); nursing students at the first, second, third, fourth and fifth academic level (**d**); nursing students with excellent, very good, good and acceptable academic performance (**e**); and nursing students with a high, moderate, low and poor level of knowledge on organ donation (**f**). Bars represent the mean ± SD. * *p* < 0.05, ** *p* < 0.005, *** *p* < 0.001.

According to the one-way ANOVA test, significant differences were observed among nursing students regarding their overall knowledge scores (F(2,275) = 15.07, *p* < 0.001). Nursing students who were willing to donate their organs showed significantly higher overall knowledge scores (7.33 ± 3.23) compared to those who were not willing to donate their organs (5.21 ± 3.09, *p* <0.001), as indicated by Tukey’s post hoc test. Regarding favorable attitude, unfavorable attitude, and distrust scores, significant differences were observed between the groups, which were determined using Kruskal–Wallis H tests at χ^2^ (2) = 61.58, *p* < 0.001, χ^2^ (2) = 75.06, *p* < 0.001 and χ^2^ (2) = 32.66, *p* < 0.001, respectively. Nursing students who were willing to donate their organs had a significantly higher favorable attitude (4.55 ± 0.60, *p* < 0.001) and lower unfavorable attitude (2.35 ± 0.91, *p* < 0.001) and distrust scores (2.02 ± 0.81, *p* < 0.001) compared to nursing students who were not willing to donate their organs (5.21 ± 3.09, 2.94 ± 0.68 and 2.97 ± 0.81, respectively) (Figure 1).

#### 3.4.2. Donor Registration

Nursing students who were already registered donors had significantly higher overall knowledge and favorable attitude scores and a lower unfavorable attitude and distrust scores (8.52 ± 2.99, *p* < 0.001, independent sample *t*-test; 4.64 ± 0.53, U = 9616, *p* < 0.001; 1.90 ± 0.78, U = 3857, *p* < 0.001 and 2.14 ± 0.92, U = 4132, *p* < 0.001, respectively, Mann–Whitney U test) than nursing students who were not registered donors (5.80 ± 3.17, 4.13 ± 0.81, 2.55 ± 0.86 and 2.74 ± 0.87, respectively) (Figure 1).

#### 3.4.3. Donation Medal

A one-way ANOVA revealed significant differences between the donation medal groups regarding knowledge and distrust (F(3,274) = 15.58, *p* <0.001 and F(3,274) = 9.02, *p* <0.001, respectively). Nursing students with a gold donation medal had higher overall knowledge scores (9.40 ± 2.47, *p* <0.001) and lower distrust scores (1.98 ± 0.83, *p* <0.001) compared to nursing students without a donation medal (5.80 ± 3.17 and 2.74 ± 0.87, respectively, Tukey post hoc test). Additionally, the Kruskal–Wallis H test showed significant differences between the donation medal groups for favorable (χ^2^ (3) = 25.84, *p* < 0.001) and unfavorable attitudes (χ^2^ (3) = 30.87, *p* < 0.001). Nursing students with a gold donation medal had higher favorable attitude scores (4.69 ± 0.41, *p* <0.001) and lower unfavorable attitude scores (1.77 ± 0.74, *p* <0.001) compared to nursing students without a donation medal (4.13 ± 0.81 and 2.55 ± 0.86, respectively, Dunn–Bonferroni post hoc test) (Figure 1).

#### 3.4.4. Academic Level

A significant difference in overall knowledge scores between academic levels was observed using a one-way ANOVA (F(4,273) = 5.26, *p* < 0.001). Nursing students at the fifth (7.24 ± 3.64, *p* = 0.02) and fourth (7.37 ± 2.92, *p* = 0.008) academic levels had significantly higher knowledge scores than nursing students at the second academic level (5.30 ± 3.35), as indicated by Tukey’s post hoc test. There was also a significant difference in favorable attitude scores between academic levels, as determined using the Kruskal–Wallis H test, χ^2^ (4) = 11.65, *p* = 0.02. Nursing students at the fourth level (4.52 ± 0.57) rated themselves as having a significantly higher favorable attitude than nursing students in the fifth (4.20 ± 0.71, *p* = 0.023), third (4.21 ± 0.76, *p* = 0.036) and first (3.98 ± 0.90, *p* = 0.002) academic levels (Dunn–Bonferroni post hoc test). Nursing students at the second academic level (4.27 ± 0.89, *p* = 0.041) rated themselves as having significantly higher favorable attitudes than nursing students at the first academic level (3.98 ± 0.90), as suggested by the Dunn–Bonferroni post hoc test (Figure 1).

#### 3.4.5. Academic Performance

Regarding academic performance, only favorable attitude scores were significantly different among the groups (χ^2^ (3) = 10.58, *p* = 0.01, Kruskal–Wallis H test). Nursing students with excellent (4.73 ± 0.79, *p* = 0.002) and very good (4.24 ± 0.78, *p* = 0.019) academic performance had significantly higher favorable attitudes than nursing students with acceptable academic performance (3.96 ± 0.83), as indicated by the Dunn–Bonferroni post hoc test (Figure 1).

#### 3.4.6. Level of Knowledge on Organ Donation

According to the Kruskal–Wallis H test, the level of knowledge about organ donation significantly influenced the development of attitudes toward organ donation (χ^2^ (3) = 33.91, *p* < 0.001). Nursing students with a high level of knowledge of organ donation had significantly higher favorable attitudes (4.58 ± 0.63) than nursing students with moderate (4.28 ± 0.69, *p* = 0.003), low (4.19 ± 0.65, *p* < 0.001), and poor (3.73 ± 0.97, *p* < 0.001) knowledge of organ donation. They also demonstrated lower unfavorable attitudes (1.91 ± 0.84) and distrust (2.15 ± 0.89) than nursing students with moderate (2.43 ± 0.87, *p* < 0.001 and 2.67 ± 0.92 *p* < 0.001, respectively), low (2.65 ± 0.83, *p* < 0.001 and 2.86 ± 0.83 *p* < 0.001, respectively), and poor (2.89 ± 0.62, *p* < 0.001 and 2.96 ± 0.75 *p* < 0.001, respectively) knowledge of organ donation. Nursing students with moderate knowledge of organ donation had significantly higher favorable attitudes (4.28 ± 0.69) than nursing students with a poor knowledge of organ donation (3.73 ± 0.97, *p* < 0.002). They also showed lower unfavorable attitudes and distrust (2.43 ± 0.87 and 2.67 ± 0.92, respectively) than nursing students with poor knowledge of organ donation (2.89 ± 0.62, *p* = 0.001 and 2.96 ± 0.75 *p* = 0.030, respectively) (Figure 1).

Religious belief was previously reported to be the leading cause of refusal to donate [1]. Therefore, examining this factor among participants in the present study revealed a significant relationship between nursing students’ religious belief (indicated by item K6) and their organ donation status χ^2^ (2) = 9.074, *p* = 0.011 using the Chi-Square test. The majority of nursing students (66.7%) who believed that Islam opposes organ donation did not donate their organs. However, there was no relationship between nursing students’ religious belief and their willingness to donate their organs.

## 4. Discussion

This cross-sectional comparative study aimed to assess the knowledge and attitudes of nursing students toward organ donation, explore the factors affecting their knowledge and attitudes, and examine the impact of knowledge on their attitudes. It was found that nursing students’ knowledge of organ donation was relatively low, with an average score of 6 out of 15. In contrast, the majority of nursing students were in favor of organ donation. In addition, several factors were positively associated with the knowledge and attitudes of nursing students, including their willingness to donate, donor registration, and donation medals based on their number and type of donated organs, academic level, academic performance, and knowledge level.

The low level of nursing students’ knowledge of organ donation is consistent with previous studies conducted among nursing students in Turkey [21], medical students in the United Kingdom and Saudi Arabia [22,23], and medical and nursing students in Mexico [19]. Similarly, their favorable attitude toward organ donation is inconsistent with several previous cross-sectional studies conducted among nursing students in Mexico, Italy, and India [19,24,25]. Although these countries have different cultures and religions, they yielded similar findings. This suggests that knowledge might have a stronger impact on nursing students’ attitudes toward organ donation when compared with religions and cultures. In addition, nursing students with low knowledge levels, despite having favorable attitudes, might be due to unstructured teaching regarding organ donation, which may affect healthcare practices, such as organ donation awareness.

Additionally, high knowledge scores and favorable attitudes toward organ donation increased students’ willingness to donate. This finding is consistent with data from a study conducted among medical and nursing students in Mexico [19]. This indicates that providing structured training at the university level might improve students’ knowledge and positive attitudes toward organ donation and might also increase the number of potential donors among future healthcare providers. Indeed, students who received previous organ donation training had higher knowledge scores than those who did not receive training [19]. A pretest–posttest study conducted among nursing students in Turkey showed that structured training increased knowledge scores and favorable attitudes toward organ donation [21].

Questionnaire analyses highlighted gaps in the knowledge among nursing students. Although most nursing students correctly answered questions related to the types of donors, more than half of them misunderstood questions relating to organ donation from brain-dead donors. Approximately 65% of nursing students did not fully comprehend that brain death was irreversible, which is consistent with similar studies among medical and nursing students in Mexico and Korea [18,19]. In addition, medical students in previous studies were unable to identify the physicians responsible for determining whether the patient was brain dead [26] or misunderstood questions related to brain-stem death testing criteria [22]. These findings suggest the need to emphasize organ donation from brain-dead donors in nursing courses.

Furthermore, the current study found that students who were organ donor card holders demonstrated higher knowledge scores and more favorable attitudes toward organ donation compared to unregistered donor nursing students. Of these, nursing students who agreed to donate all their organs (gold medal card holders) demonstrated even higher knowledge scores and more favorable attitudes toward organ donation than those who agreed to donate two or fewer organs. Although approximately 57% of nursing students expressed a willingness to donate and showed favorable attitudes toward organ donation, only 23% were organ donor card holders. In addition, approximately 14% of nursing students agreed to be gold medal donors. Similar findings were reported among medical students, with more than half demonstrating favorable attitudes toward organ donation. However, only 25% of them were officially registered as organ donors, and only 3.4% had registered and donated organs in Mexico and Turkey, respectively [19,26]. These findings indicate a disparity between favorable attitudes toward organ donation and actual practice. It is possible that some students may have provided socially acceptable responses (reporting bias) to questions regarding their attitudes [27]. It is also possible that some students fear that healthcare providers do not respect their corpses, or they fear the surgical removal of vital organs after death. This disparity between attitudes and practices suggests the need to assess knowledge and attitudes and their association with students’ actual practices, such as registration and organ donation.

The academic performance of nursing students was found to only predict favorable attitudes toward organ donation. Specifically, students with excellent and very good academic performance demonstrated more favorable attitudes toward organ donation than nursing students with acceptable and good academic performance. This novel finding is worthy of further investigation since it has not been reported in previous studies. Regarding the students’ year of study, the findings revealed that bachelor students’ knowledge of organ donation increased as they progressed to their fourth and fifth (internship) years of study. It is possible that students need more time to comprehend the knowledge related to organ donation, which explains the knowledge level difference among academic years. However, although the year of study predicted favorable attitudes toward organ donation, students’ attitudes did not improve as they progressed to the internship year compared to their fourth year of study. Similarly, in Turkey, medical students’ knowledge of organ donation improved as they progressed from their first to sixth year; however, students’ attitudes and behaviors did not favor organ donation [26]. These findings indicate a lack of structured clinical training in transplant units. Structured lectures combined with clinical training in transplant units could enhance empathy toward patients and their families, consequently leading to more favorable attitudes toward organ donation as students progress to their final years of study.

Additionally, the level of knowledge about organ donation significantly influences the development of attitudes toward organ donation. Nursing students with higher levels of organ donation knowledge demonstrated significantly more favorable attitudes than nursing students with moderate, low, or poor knowledge. These results are consistent with previous studies conducted on both nursing and medical students, showing that higher knowledge scores on organ donation are positively correlated with more favorable attitudes toward organ donation [19]. According to two systematic reviews, participants’ knowledge, attitudes, and behaviors were significantly transformed by education on organ donation and transplantation [28]. Formal education on organ donation has been found to positively influence student nurses’ attitudes, promote better communication and registration behaviors, and improve their understanding of donor eligibility and brain death [29].

### 4.1. Limitations

Although the present study reveals important findings, it has some limitations. One limitation is that the present study used a cross-sectional comparative design, which only revealed a description of the knowledge and attitude of nursing students toward organ donation. However, it did not address educational interventions that could enhance the knowledge and attitude of this population. Also, the study was performed in one university that included female nursing students only. Therefore, these data may have limited generalizability. Extending these investigations into male nursing students and other nursing students in other Saudi universities may provide better insights on the subject. Moreover, the use of non-probability convenience sampling may not be representative of the entire population. In addition, it is possible that students may have reported what they perceived to be socially desirable. Nevertheless, the present study provides valuable information pertaining to nursing students’ practice, particularly their registration status and its association with their knowledge and attitudes toward organ donation.

### 4.2. Practical and Policy Implications

The findings of this study have practical implications for nursing education and healthcare practice. Nursing academic leaders may take steps to improve nursing students’ knowledge and attitudes toward organ donation. They can remove considerable obstacles and promote prosocial behavior by disseminating knowledge about organs that are acceptable for donation, the mechanisms for registration, and the relevant laws in Saudi Arabia [30]. Disseminating knowledge regarding laws is important as recently, in 2021, the laws have changed to include policies regarding circumstances prohibiting organ donation, examining the human organ, the dignity of the donor and protecting the donor from humiliation or mutilation, and verifying death for the purpose of donating organs. Considering that the nursing education curricula do not typically include instructions on organ donation and transplantation practices, it is important for nursing students to receive additional systematic structured knowledge to encourage more nursing students to become registered organ donors and increase the number of potential donors among future healthcare providers.

### 4.3. Future Direction

These findings indicate the significance of prioritizing organ donation research to identify and address potential barriers to donation, particularly in light of the disparity between organ availability and the demand for transplantation. Given the findings and limitations of the current study, future research is needed to investigate the topic using a multi-site, pretest–posttest, control group design. In addition, it is recommended to include both female and male nursing students.

## 5. Conclusions

This cross-sectional comparative study aimed to assess the knowledge and attitudes of nursing students toward organ donation, as well as the factors influencing their knowledge and attitudes, including the impact of knowledge on their attitudes. The study revealed that nursing students exhibited a relatively low level of knowledge regarding organ donation. On the contrary, a significant proportion of nursing students showed a positive attitude toward organ donation. Furthermore, several factors exhibited a positive correlation with the knowledge and attitudes of nursing students. These factors included their willingness to donate their organs, donor registration, donation medals, academic performance, academic level, and level of knowledge. This study is novel because, to the best of our knowledge, it is the first of its kind to explore the specific factors pertaining to nursing students’ practices, such as their registration status and the type of organ donation medal they received. Both the academic performance and knowledge level of nursing students on organ donation positively impact their attitude toward organ donation. This signifies the need to modify nursing education curricula to include lectures on organ donation and transplantation practices. Further research is necessary to identify effective strategies that enhance the knowledge and attitude of nursing students toward organ donation. Improving knowledge of organ donation through nursing curricula and research could potentially increase the number of donors among future nursing students and, by extension, the broader population.

## Figures and Tables

**Table 1:** Demographic characteristics.

Variable	n (%)
Nursing student (n.)	278
Age range (years)	
18–22	239 (86%)
23–27	35 (12.6%)
28+	4 (1.5%)
Academic level	
First year	52 (18.7%)
Second year	50 (18%)
Third year	59 (21.2%)
Fourth year	62 (22.3%)
Fifth year (internship)	55 (19.8%)
Academic performance	
Excellent	81 (29.1%)
Very good	148 (53.2%)
Good	36 (12.9%)
Acceptable	13 (4.7%)
Organ donation training	
Yes	4 (1.4%)
No	274 (98.6%)
Willingness to donate	
Yes	160 (57.6%)
No	112 (40.3%)
I do not know	6 (2.2%)
Donor registration	
Registered	64 (23%)
Non-registered	214 (77%)
Donation medal	
Gold	40 (14.4%)
Silver	13 (4.7%)
Bronze	11 (4%)
None	214 (77%)

**Table 2:** Participants’ knowledge about organ donation (frequencies of correct/incorrect answers).

Item	I Do Not Know	Incorrect n (%)	Correct n (%)
1. Mark the types of donors that exist:			
1.a. Living donor *	-	95 (34.2%)	183 (65.8%)
1.b. Brain-dead donor *	-	77 (27.7%)	201 (72.3%)
1.c. Circulatory death donor *	-	76 (27.3%)	202 (72.7%)
2. Among the types of donors you marked, from which can the most organs be extracted? 1.a. Living donor 1.b. Brain-dead donor * 1.c. Circulatory death donor	-	151 (54.3%)	127 (45.7%)
3. Mark the organs or tissues that can be transplanted:			
3.1. Skin.	-	94 (33.8%)	184 (66.2%)
3.2. Bone.	-	144 (51.8%)	134 (48.2%)
3.3. Liver.	-	42 (15.1%)	236 (84.9%)
3.4. Pancreas.	-	97 (34.9%)	181 (65.1%)
3.5. Cochlea. *x*	-	207 (74.5%)	71 (25.5%)
3.6. Blood vessels.	-	152 (54.7%)	126 (45.3%)
3.7. Heart.	-	185 (66.5%)	93 (33.5%)
3.8. Brain. *x*	-	84 (30.2%)	194 (69.8%)
4. There are people on waiting lists for a transplant who die because there are not enough organs available.	25 (9%)	5 (1.8%)	248 (89.2%)
5. Organ or tissue donation disigures the body in such a way that it is not possible to perform a funeral with the coffin open. *x*	83 (29.9%)	47 (16.9%)	148 (53.2%)
6. Religions derived from islam oppose organ and tissue donation. *x*	104 (37.4%)	54 (19.4%)	120 (43.2%)
7. Brain death is the irreversible cessation of all brain function, including brainstem.	59 (21.2%)	36 (12.9%)	183 (65.8%)
8. Brain death is a legally recognized death criterion.	82 (29.5%)	61 (21.9%)	135 (48.6%)
9. There are people who have recovered from brain death. *x*	117 (42.1%)	65 (23.4%)	96 (34.5%)
10. Organ transplantation is viable only among family members.	24 (8.6%)	17 (6.1%)	237 (85.3%)
11. There is an age limit to donating organs and tissues. *x*	63 (22.7%)	97 (34.9%)	118 (42.4%)
12. Having a cardiovascular disease prevents being an organ donor. *x*	135 (48.6%)	79 (28.4%)	64 (23.0%)

* Correct choice. *X* Incorrect statement.

**Table 3:** Participants’ knowledge levels and attitude scores.

	Variable	Mean ± SD
Knowledge	Global score (out of 15)	6.43 ± 3.33
	High	10.24 ± 1.30
	Moderate	6.89 ± 0.83
	Low	4.57 ± 0.50
	Poor	1.46 ± 1.57
Attitude	Favorable Attitude	4.25 ± 0.78
	Unfavorable Attitude	2.40 ± 0.88
	Distrust Attitude	2.60 ± 0.92

## Data Availability

Data generated as part of this study are available from the corresponding author on reasonable request.
